# The effect of processing load on loss of information from short-term memory

**DOI:** 10.1080/09658211.2018.1497661

**Published:** 2018-07-12

**Authors:** Dennis Norris, Jane Hall, Sally Butterfield, Michael P.A. Page

**Affiliations:** a MRC Cognition and Brain Sciences Unit, Cambridge, UK; b Department of Psychology, University of Hertfordshire, Hatfield, UK

**Keywords:** Memory, short-term memory, Stroop, forgetting

## Abstract

We report an experiment in which we varied the nature of the articulatory suppression task being performed during a filled retention interval in serial recall. During the retention interval participants performed one of three computer-paced colour naming tasks designed to prevent subvocal rehearsal: A Stroop color-interference task with items presented at a rate of one every 750 ms, and two color-consistent control tasks at a rate of either 750 ms or 500 ms per item. Memory performance over a 12 s interval declined much more dramatically with the Stroop task and the 500 ms control task than with the 750 ms control. There was no difference between the Stroop condition and the 500 ms control. These results pose problems for models that assume that loss of information from memory is determined entirely by interference, as there are more interfering events in the control 500 ms condition than the 750 ms Stroop. They also pose problems for models relying solely on time-based decay and articulatory rehearsal because all three conditions should block rehearsal and produce equivalent performance. The results illustrate that articulatory suppression tasks are not all equivalent, and suggest that the rate of decay from short-term memory is strongly influenced by the resource demands of concurrent processing

When rehearsal is prevented, memory for as few as three letters drops to chance over a period of about 20 s (Brown, 1958; Peterson & Peterson, 1959). Why should this be? The two most commonly offered interpretations of this finding have been that memory undergoes a time-based decay that can be prevented if the memory trace is continually refreshed by rehearsal, or that events taking place during the retention interval interfere with the representations of items in memory. The question of whether forgetting is best characterised as interference or decay is still a matter of active debate (Barrouillet, Uittenhove, Lucidi, & Langerock, 2017; Lewandowsky & Oberauer, 2015; Lucidi et al., 2016; Oberauer, Farrell, Jarrold, & Lewandowsky, 2016; Ricker, Spiegel, & Cowan, 2014; Vergauwe, Camos, & Barrouillet, 2014). Indeed, McGeoch’s (1932) view that “There are no warmly discussed theories of forgetting” might still be echoed today.

One of the main reasons for the intractability of the question is that the main factors that have been proposed to account for forgetting are generally highly correlated; the passage of time, the unfolding of events, and the duration of processing. Here we examine forgetting specifically in the context of short-term verbal memory. We report a simple experiment which poses a particular challenge to all current theories of forgetting from short-term memory.

The most influential example of a theory of short-term memory where forgetting is due to decay is the Working Memory model of Baddeley and Hitch (Baddeley, 1986; Baddeley & Hitch, 1974). Their model assumes that forgetting is the result of decay, but the contents of memory can be refreshed by sub-vocally rehearsing items using the Articulatory Loop subsystem. According to this view, if rehearsal is prevented by blocking the operation of the loop, forgetting should occur. However, this simple story requires extensive qualification. In particular, there is no straightforward way to determine what kind of activity will prevent rehearsal. For example, Vallar and Baddeley (1982) found that whereas counting backwards in threes seemed effective in blocking rehearsal, in that it lead to rapid forgetting, simply repeating “the” produced hardly any forgetting at all, even though one might expect continuous articulation to block the operation of the loop. Although it is possible that counting backwards in threes occupies the loop more fully than repeating “the”, it is also possible that backwards counting is more deleterious because it imposes a greater processing load. One of the first indications that forgetting might depend on the difficulty of the task performed during retention came from Posner and Rossman (1965) who reported that forgetting was a function of the amount of information reduction required by the task. Their results are consistent with resource models of working memory where there is a pool of resources that has to be shared between maintenance and any processing operations carried out during the maintenance period (e.g., Just & Carpenter, 1992; Ma, Husain, & Bays, 2014). However, others have suggested instead that more demanding tasks take more time and leave less time for maintenance processes. That is, demanding tasks are not detrimental because of the load they impose but because of the time they take. For example, Towse, Hitch, and Hutton (2002) argued for a simple task-switching model where resources could be switched between the retention task and maintenance. They assumed that forgetting was determined purely by the amount of time taken up by the retention task, and not by its difficulty. However, in their experiments, task difficulty and duration were confounded. Furthermore, the retention-interval tasks were all self-paced. Participants may have adapted to the “harder” tasks by continuing to use resources at the same rate, but for a longer period. Barrouillet and colleagues (Barrouillet, Bernardin, & Camos, 2004; Barrouillet, Bernardin, Portrat, Vergauwe, & Camos, 2007) addressed some of these issues by using computer-paced secondary tasks. They argue that the impact of having to perform a secondary task during retention is a function of the duration of the task which competes for time devoted to maintenance.

In common with Cowan and colleagues (e.g., Cowan, 1992) they suggest that maintenance of the memory trace may not require the kind of explicit sequential rehearsal process envisaged in the Working Memory model. Instead, there may be a rapid non-articulatory refreshing process. The Barrouillet studies used variations on an operation span task in which the secondary task is interleaved between presentations of successive items to be recalled. In their studies the retention interval for the first item can be up to one minute. In the Working Memory model, retention over such long delays would be considered to be served by the Episodic Buffer (2000) rather than the short terms stores, and the necessary task switching would require extensive coordination by the Central Executive. This makes it hard to compare their results with those from more conventional simple-span serial recall tasks where items have to be retained for only a few seconds. Indeed, very little of the recent work on the nature of forgetting has studied short-term verbal memory. For example, the bulk of the work covered by Oberauer et al. (2016) in their recent review used either visual working memory tasks or complex span tasks.

Models incorporating interference can also explain why memory declines during filled maintenance periods. In these models, memory deteriorates as a function of the number of interfering items incorporated into memory, and not because of time-based decay. For example, Lewandowsky and Oberauer (2015) have argued that not only is there no decay in short-term memory, but also that articulatory rehearsal is no benefit. Their general line of reasoning is that rehearsal can introduce errors, and that every rehearsal episode will therefore tend to introduce more errors and make performance deteriorate. However, while this may be a property of the specific implementation of a decay model they chose to use (LTRS*, Oberauer & Lewandowsky, 2011) this is not true of all decay models. For example, Page and Norris (1998) incorporated rehearsal into their Primacy model. In that model participants are assumed to rehearse while they are still able to repeat the items encountered so far in the interval between the presentation of successive items. For example, they might rehearse only up until the fourth item has been presented. The items up until that point will have undergone very little decay and rehearsal will be completely accurate. This is exactly the same process that enables short lists of items to be retained indefinitely when participants are permitted to rehearse. Of course, the same is not true when lists exceed span. For example, an eight-item list is unlikely to be recalled without error. Any attempt to repeatedly rehearse entire eight-item lists is likely to lead to errors that propagate and increase with successive rehearsals. This process can be seen at work in the Hebb (1961) repetition task. Kalm and Norris (2016) found that, as would be expected, performance on immediate serial recall of an eight-item list improved over successive repetitions, but errors tended to be similar to errors in recall of previous lists. That is, when there are errors in recall these do propagate through subsequent recalls, even when participants have the benefit of a repeated presentation of the list in between.

There are thus several alternative accounts of why information is lost from memory over time, and why that loss is greater when participants engage in a task such as articulatory suppression during retention. According to the standard Working Memory account, loss of information from STM is a consequence of decay which can be counteracted by articulatory rehearsal. In resource models maintenance depends on sharing a pool of resources between maintenance and other processing requirements. In Barrouillet et al.’s (2004, 2007) Time-Based Resource-Sharing (TBRS) modelTBRS modelthe resource is not shared simultaneously, but switched rapidly between either maintenance or processing. In TBRS the measure of resource demands is processing time, Tasks are more resource demanding to the extent to which they take more time to process. Finally, in strict interference models, loss of information is a function purely of the number of interfering events.

One task that has frequently been used to prevent rehearsal during a retention interval is digit shadowing. The rate of presentation of the digits has varied between one every 400 ms (Bjork & Healy, 1974) to one every 750 ms (Norris, Baddeley, & Page, 2004). Subjectively at least, digit shadowing, at even the 750 ms rate of presentation, appears to prevent conscious sequential rehearsal of the list items. Norris et al. reported that they had never encountered a participant who claimed to be able to rehearse while shadowing. A shadowing task where processing load and number of potentially interfering items could be manipulated without changing the fundamental nature of the task would therefore provide an ideal way of examining the effect of load on memory. Here we vary load using a Stroop colour naming task (Stroop, 1935). Participants are presented with a series of colour names printed in colour and the task is to name the ink colour. In the “easy” color-consistent version of the task, the colour word describes the colour of the ink. The “hard” version of the task uses the standard Stroop manipulation where the colour word corresponds to a different colour from the ink (e.g., GREEN printed in red). The Stroop manipulation ensures that the easy and hard conditions are completely equated for the perceptual characteristics of the input (coloured words) and the responses required. Any differences between conditions are therefore unlikely to be attributable to differential effects of interference. Barrouillet, Portrat and Camos (2011) also used a Stroop task as a distractor in one of their studies of working memory. Performance in the Stroop condition was impaired relative to a neutral control. However, this was a complex span task where the retention interval for the first digit in a six item sequence could be almost a minute. Each digit to be remembered was followed by eight colour or neutral words where participants had to read the colour of the ink aloud. Each digit was presented at a slow rate (1.5 s followed by a 0.5 s blank) and the coloured words were presented at a rate of one per second. Here we use a simple serial recall task and track the effect of the Stroop distractor from 0.5–12 s.

Preliminary investigations indicated that the fastest rate that participants could reliably perform the colour naming was about 750 ms in the Stroop condition and about 500 ms in the consistent condition. We therefore set the standard presentation rate to be one item every 750 ms. Although, given the reports from (Norris et al., 2004), we were fairly confident that participants would not be able to perform a full sequential rehearsal of the list items, in the color-consistent condition it is conceivable that some time might be available to perform a partial rehearsal. We therefore also included a second control condition where colours were presented at a rate of one every 500 ms. If all three conditions are equally successful at preventing rehearsal then, according to the Working Memory model, the decline in memory performance over time will be the same in all cases. However, according to an interference account, memory should decline as a simple function of the number of interfering items. Performance should therefore be equivalent in the Stroop 750 ms and the 750 ms control conditions, but worse in the 500 ms control condition. In contrast, if the Stroop condition is more resource demanding, it should be harder than the 750 ms control, possibly being equivalent to the 500 ms control. We also included a manipulation of phonological confusability to give an index of whether the letters were held in the phonological store at the different retention intervals.

## Experiment

Participants were 54 members of the Cognition and Brain Sciences Unit volunteer panel aged between 16 and 25 years. There were equal numbers of males and females. There were 18 participants in each of the Stroop and 750 ms and 500 ms control conditions

The Stroop condition and the 750 ms rate colour consistent control conditions both had exactly the same timing and differed only in terms of the colours the words were printed in. On each trial participants heard a list of four consonants presented over headphones at a rate of one letter every 750 ms. The consonants were spoken in a male voice, and had been edited so that they sounded evenly paced no matter what order they appeared in. In both the Stroop condition and the 750 ms color-consistent control, 750 ms after the onset of the last consonant participants either saw the word “recall”, or the word “loud” followed by three or fifteen colour words, and then the word “recall”. The word “loud” reminded participants to switch to reading the colours aloud. When the word “recall” appeared, participants had to write the letter list in the correct order in response boxes provided. All of the visual stimuli were presented for 650 ms with a 100 ms gap between them. This timing arrangement meant that there were three retention intervals of 0.75 s, 3 s, and 12 s. Trials with different retention intervals were randomly intermixed.

In the 500 ms rate color-consistent condition, all visual stimuli were presented at a rate of one every 500 ms (450 ms display plus 50 ms blank), and there were zero, five, or 23 colour words, giving retention intervals of 500 ms, 3 s, and 12 s. All participants received 6 practice trials followed by 144 experimental trials. There was a short break half way through the experiment.

The colour words were randomly sampled from the set RED, GREEN, YELLOW, BLUE, all presented in upper case. The same word never appeared twice in succession. In the color-consistent control condition, colour words were displayed in the colour corresponding to the word. In the Stroop condition, the word was always displayed in a different colour. Words were presented in a 16 point bold Arial font on a 19 inch CRT monitor.

The consonant lists were all constructed from the sets ZJHR, PBLD and SXQF. The set ZJHR contains only phonologically non- confusable letters, whereas the other two lists contain three confusable letters and one nonconfusable letter. Overall there were therefore equal numbers of confusable and nonconfusable letters.

### Results

**Figure 1.**
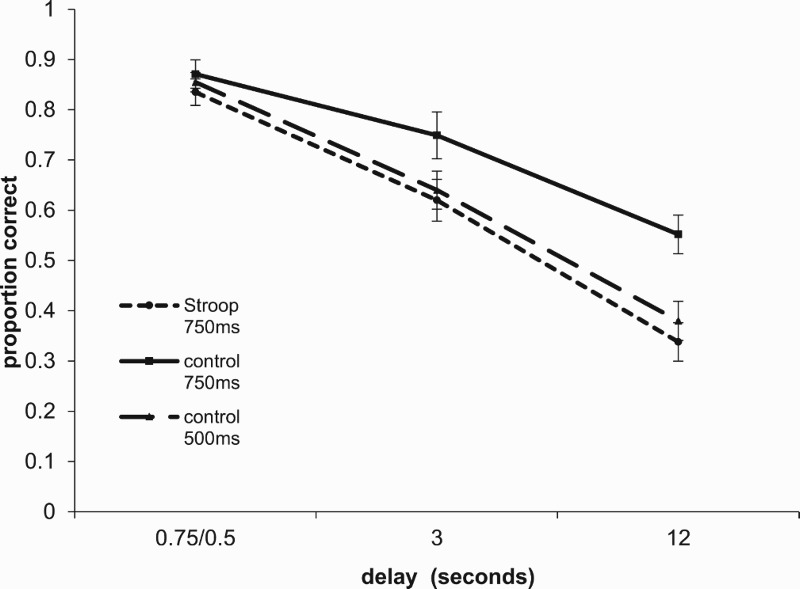
Mean proportion of items correct in position at different retention intervals for the Stroop condition presented at 750 ms, and for the consistent condition at 750 ms and 500 ms rates. Error bars are ±1 standard error.

Recall was scored in terms of number of items recalled in the correct position. Recall scores are presented in Table 1 and are plotted in Figure 1. The primary interest here is in the comparison between the Stroop condition and the 750 ms control. This was analyzed with a 2 (task) × 2 (confusability) × 3 (delay) × 4 (serial position) repeated measures ANOVA. All reported effects are significant at the .05 level. There was a main effect of task, *F*(1,34) = 6.12, $\eta_{p}^{2}=\;.154$, delay, *F*(2,68) = 159.74, $\eta_{p}^{2}=\;.825$, with memory in the Stroop condition being worse than in the consistent condition. Confusable items were recalled significantly less accurately than nonconfusable items, *F*(2,34) = 38.43, $\eta_{p}^{2}=\;.531$, and there was a main effect of serial position *F*(3, 102) = 47.72, $\eta_{p}^{2}=\;.584$. The interaction between task and delay was significant *F*(2,68) = 7.48, $\eta_{p}^{2}=\;.180$, with the effects of task (Stroop vs. consistent) being greater at longer delays. There was also an interaction between confusability and delay, *F*(2,204) = 15.82, $\eta_{p}^{2}=\;.318$, reflecting the fact that the effects of phonological confusability decreased over time, and confusability and serial position, *F*(3,204) = 16.43, $\eta_{p}^{2}=\;.326$. Planned comparisons showed that the effect of task was significant at both the 3 s, *F*(1,34) = 4.245, $\eta_{p}^{2}=\;.11$, and 12 s delays, *F*(1,34) = 10.276, $\eta_{p}^{2}=.232$, but not at the .75 s delay (*F* < 1).

**Table 1.** Mean proportion of items correct in position.

	confusibility	Delay			Mean
		.75/.5	3 s	12 s	
Stroop 750 ms	nonconfusable	.90	.67	.34	.64
	confusable	.77	.57	.33	.56
Consistent 750 ms	nonconfusable	.93	.81	.57	.77
	confusable	.81	.69	.53	.67
Consistent 500 ms	nonconfusable	.93	.70	.38	.67
	confusable	.78	.58	.38	.58

A second analysis compared the 750 ms and 500 ms control conditions. There were main effects of rate, *F*(1,34) = 4.41, $\eta_{p}^{2}=.115$, delay, *F*(2,68) = 155.48, $\eta_{p}^{2}=.821$, confusability, *F*(2,34) = 50.83,$\;\eta_{p}^{2}=.599$, and serial position, *F*(3, 102) = 66.11, $\eta_{p}^{2}=.660$. There were also significant two-way interactions between rate and delay, *F*(2,68) = 6.06, $\eta_{p}^{2}=.151$, confusability and delay, *F*(2,204) = 18.19, $\eta_{p}^{2}=.349$, confusability and serial position *F*(3,204) = 17.47, $\eta_{p}^{2}=\;.339$, and delay and position, *F*(6,204) = 5.24, $\eta_{p}^{2}=\;.134$. Additionally, there were significant three way interactions between delay, confusability and serial position, *F*(6.204) = 3.55, $\eta_{p}^{2}=.094$, and delay, rate, and serial position *F*(6,204) = 3.40, $\eta_{p}^{2}=.091$. Planned comparisons showed that the effect of task was significant at the 12 s delay, *F*(1,34) = 6.605, $\eta_{p}^{2}=.163$, but not at 3 s, *F*(1,34) = 3.755, *p* = .061, $\eta_{p}^{2}=.099$.

The effect of phonological confusability was analyzed separately for all three task conditions at all three delays. For all conditions memory for confusable items was significantly worse than for non-confusable items at the two shorter delays but not at the 12 s delay (Stroop 750: *F*(1,17) = 23.12, $\eta_{p}^{2}=.576$; 3 s *F*(1,17) = 14.62, $\eta_{p}^{2}=\;.462$; 12 s, *F*(1,17) < 1; Consistent 750: *F*(1,17) = 30.40, $\eta_{p}^{2}=.641$; 3 s *F*(1,17) = 30.07, $\eta_{p}^{2}=\;.639$; 12 s, *F*(1,17) = 1.65; Consistent 500: 0.5 s, *F*(1,17) = 47.53, $\eta_{p}^{2}=.737$; 3 s *F*(1,17) = 16.06, $\eta_{p}^{2}=\;.486$; 12 s, *F*(1,17) < 1). In a final analysis comparing the Stroop condition and the 500 ms control condition, there was no main effect of task (*F* < 1) and there were no significant interactions involving task

## Discussion

In discussing the idea of decay as represented by Thorndike’s (1913) *law of disuse*, McGeoch (1932) pointed out that “It is virtually impossible to vary the period of disuse without varying also the number or kind, or both, of the conditions or events which fill that period” (p. 361). The Stroop task allows us to break the link between time and number of events so as to directly contrast the effects of interference and decay. We can examine memory over a given period while varying the number of potentially interfering items. In our experiment, all three conditions lead to rapid forgetting over a 12 s delay. Importantly, forgetting was far greater in both the Stroop condition and the 500 ms consistent condition than in the 750 ms consistent condition. For the Stroop condition this was true even at the 3 s retention interval. The first point these data make is that the exact choice of task used in the retention interval can have a dramatic effect on the rate of forgetting. After 12 s, performance on the Stroop condition was at only 61% of the level attained in the 750 ms control condition. This is true despite the fact that, subjectively at least, even the 750 ms control condition seems to allow no time for conscious sequential rehearsal of the list items. Second, the Stroop and the 750 ms color-consistent conditions are precisely controlled for the representations involved in performing the task. All that varies between them is the pairing of colours and colour words. This means that it is highly unlikely that the extra forgetting observed in the Stroop condition can be attributed to extra interference. These data seem to rule out both an interference account and an account based purely on decay that can be offset by articulatory rehearsal.

It might seem possible to rescue a pure interference theory by arguing that each Stroop word has both an ink colour and a colour name, whereas a color-consistent word has only one colour. Perhaps the Stroop items might need to be encoded using more features and this might generate more interference? Such a view would run into at least two problems. The first is that it is unclear why features used to represent ink colour would interfere with the representations of letters stored in STM. How could the two representations overlap in such a way that would produce interference? A second problem is that color-consistent and inconsistent words both have ink colours and colour names. The difference is simply that in the inconsistent case they are different. One would need to argue that the color-consistent words create less interference because the colour representations can be bound together in an amodal representation requiring fewer features. The difference between the two color-consistent conditions might be due to the extra interference created by having to name more colours in the faster condition. However, if there is an interference effect at work here, we would have expected to see it in the contrast between Stroop and the 500 ms color-consistent condition also. That is, both tasks should be equally effective in preventing rehearsal, but there are more interfering items in the 500 ms consistent condition than the 750 ms condition. If there is an interference effect, it is swamped by the effect of the extra difficulty of the Stroop task. Any attempt to argue that interference might provide a complete account of these data must confront a major problem with a pure interference theory – there are no a priori criteria for determining what counts as a feature, or predicting which features should interfere with each other. Indeed, even proponents of interference theory have recently come to acknowledge that this is a weakness of interference accounts and have considered the possibility that interference might need to be supplemented by resource limitations (Oberauer et al., 2016).

In order to account for these data in terms of decay and articulatory rehearsal it would be necessary to assume that when performing the 750 ms color-consistent task, there is sufficient slack time available to perform at least some rehearsal in the interval between colours. Although this would seem to be implausible, it is impossible to rule out without a categorical behavioural index of rehearsal. One important fact to note here is that there was no significant phonological similarity effect at the 12 s delay in any condition. This implies that, by this time, the phonological store was no longer playing any role in retention. If participants had been able to continue performing subvocal rehearsal using the articulatory loop and phonological store during the 12 s retention interval, information would still have been retained in the phonological store, and one would expect to see a phonological similarity effect. Although this null effect cannot rule out the possibility that there is some residual subvocal rehearsal, the data provide no evidence that any rehearsal is actually taking place within the phonological store.

The possibility that forgetting might be a consequence of decay offset by rapid attentional refreshing rather than articulatory rehearsal is certainly compatible with the data. However, the data is not what would be expected on the basis of previous estimates of the time taken to refresh items in memory. In the TBRS model, rapid attentional refreshing is assumed to take in the order of 50 ms per item (Vergauwe et al., 2014). Therefore it should have been possible to refresh our four-item lists in the 250 ms of extra time available in the 750 ms controls relative to the 500 ms controls. However, unless there is significant forgetting during the 500 ms taken to process the colour consistent word, there should have been no forgetting at all in the 750 ms control condition as it should have been possible to perform a complete refresh during every interval between words. Of course, if our participants took significantly more than 62.5 ms to refresh a single item, then not all items could be refreshed and there would be forgetting. The weakness of this account is that the rapid attentional refresh process is even more invisible than articulatory rehearsal. A similar problem arises with a more general notion of resource where processes taking the same amount of time might still vary in their resource requirements. Once the linkage between resource and time is broken, it becomes hard to predict which tasks will demand more resources and lead to more forgetting.

However, while either the simple version of the Working Memory model or some form of resource model could perhaps be forced to accord with the data, the data pose a more significant challenge to a pure interference account. A successful interference model would need to be able to specify which representations are stored in STM and how those representations might interfere with each other.
